# Disability-inclusive COVID-19 response: What it is, why it is important and what we can learn from the United Kingdom’s response

**DOI:** 10.12688/wellcomeopenres.15833.1

**Published:** 2020-04-28

**Authors:** Hannah Kuper, Lena Morgon Banks, Tess Bright, Calum Davey, Tom Shakespeare

**Affiliations:** 1 International Centre for Evidence in Disability, London School of Hygiene & Tropical Medicine, London, WC1E 7HT, UK

**Keywords:** Disability, COVID-19

## Abstract

All too often, disabled people are left behind in emergencies, and this is a risk in the ongoing COVID-19 pandemic. This is an important issue, as globally there are approximately one billion people with disabilities. This number includes one in three people aged over 60, who are the group at greatest risk from COVID-19. The COVID-19 pandemic in the UK has highlighted additional difficulties that disabled people may face. Complying with preventative measures, like social distancing, can be challenging, particular for people who rely on carers. Disabled people may also be at greater risk of morbidity and mortality if they contract the virus, yet in danger of being de-prioritised for care. Many people with disabilities have ongoing healthcare needs, and these need to still be supported during the pandemic. Furthermore, people may become newly disabled as a result of the pandemic, and therefore require appropriate care. Good practice examples have emerged for meeting these challenges, such as guidance for healthcare professionals on treating people with dementia, but these need to be scaled up further and adapted for other settings. In conclusion, it is clear that a disability-inclusive COVID-19 response is needed, both in the UK and as the pandemic unfolds globally. This response will require inclusion of disability measures within data collection, consulting with disabled people, and tailoring responses to be appropriate for this group.

## Introduction: Why is a disability-inclusive COVID-19 response important?

All too often, disabled people are left behind in emergencies, and this is a risk in the ongoing COVID-19 pandemic. This will affect many people as the World Health Organization (WHO) estimates that there are one billion disabled people globally ^1^, and 11 million within the United Kingdom (UK) alone ^2^. Globally, over a third of people older than 60 are disabled ^1^, which is the group experiencing the highest mortality rates from COVID-19. It is therefore vital to give serious consideration to the inclusion of people with disabilities in the COVID-19 response. But what does this mean, and what can we learn from the UK’s response?

## Inclusion in the direct response to COVID-19

People are being urged to physically isolate, stay at home, and reduce social contacts in order to protect themselves and others from the virus, but these measures may present difficulties for disabled people. For example, people with sensory or intellectual impairments may lack the necessary information about the virus, public restrictions and available services, if these are not provided in accessible formats. Good practice examples now exist from the UK, with information provided in British Sign Language for people with hearing loss ^3^, or in simple format for those with intellectual impairments ^4^.

Preventative measures, like social distancing or self-isolation, are often more challenging for disabled people. As an example, self-isolation is impossible if one relies on carers in daily life. Carers require information on how they can protect the health of disabled people. The Alzheimer’s Association has developed guidance for caregivers of people with dementia to explain hygienic behaviours, such as hand-washing, and suggest how to keep the person healthy and safe ^5^.

Disabled people may also be at greater risk of morbidity and mortality if they contract the virus. Not only are they older on average, but they are more likely to have underlying health conditions, such as respiratory diseases, that heighten their risk of severe morbidity and mortality if they contract the virus ^6^. The prevalence of diabetes and hypertension are co-morbidities for many disabled people ^6^, and these too are potential predictors of adverse outcomes after developing COVID-19 ^7^.

Disabled people must have equal access to quality healthcare if they develop COVID-19. Yet, knowledge and awareness about disability may be low among healthcare professionals, as this is a noted gap within their training ^8^. Doctors and nurses therefore need rapid awareness raising on disability, so, for example, they can explain to someone with cognitive impairment why they cannot see family members or what is happening to them if they contract the virus. Some relevant initiatives are underway; The NHS has produced guidance on how to include people with mental health conditions, learning disabilities and autism within the COVID-19 response ^9^. We must also ensure that disabled people will receive the same attention and quality of care as others if services become stretched and prioritisation are made. Worryingly, in the United States, some states have drafted contingency plans for the rationing of scarce resources that deprioritise people with intellectual impairments or who require assistance with activities of daily living. It is critically important to reinforce awareness that the right to health applies to all, and that it violates human rights and is morally reprehensible to deprioritise disabled people.

## Ensuring continuation of health and social services

Maintaining standards of day-to-day life may be challenging for disabled people, especially if supplies of medication or devices dwindle. In high-income settings, solutions that are available to others, such as relying on expensive home-delivery services, may be less feasible for disabled people who are on average poorer. Supermarkets in the UK are asking shoppers to register as complying with the government definition of vulnerability ^10^. While this prioritisation is welcome, the definition does not include all people in need, nor is it clear how confidentiality will be maintained. Volunteer schemes are being implemented to help fill these gaps, providing supplies to vulnerable people ^11^.

Many people with disabilities have ongoing healthcare needs. Indeed, isolation may impact particularly negatively on functioning in disabled people. People with mental health conditions can experience symptom exacerbation, and potentially a heightened risk of severe psychiatric morbidity and suicide. People with physical impairments who rely on rehabilitation therapies may have functional declines. Routine healthcare support must therefore be maintained for people with health conditions, whether this is the supply of medication, mental health support, or ongoing physiotherapy or occupational therapy (at distance). There are examples now of healthcare professionals providing consultations online, such as in China where online mental health services were provided during the epidemic ^12^. Continuation of carer support is needed for people requiring assistance in daily living, including during lockdowns or self-isolation. People whose carers are sick or self-isolating need support from care agencies in finding substitute support. The abandonment, and consequent tragic death, of older people in care homes in Spain illustrates the pressing need for guidance and oversight ^13^.

## Disability-inclusive planning and data collection

It may be clichéd, but what is not counted does not count. It is therefore critical to collect data on disability within the COVID-19 response. Disability measures should be included in data collection, so that we can track the vulnerability of disabled people to contracting the virus, becoming critically ill or dying, compared to those without disabilities. The C-19 COVID Symptom Tracker, launched by King’s College London, includes some relevant items (e.g. need for regular help, having a health problem that requires you to stay at home, and regularly using a stick, walking frame or wheelchair to get about) ^14^. These measures are welcome, but do not capture all people with disabilities, so could be enhanced by including measures of hearing, sight and cognition (e.g. Washington Group Short Set) ^15^. We must also consult with disabled people on their experiences, additional needs and suggested solutions, so that COVID-19 responses can be appropriately tailored. Large scale qualitative studies are therefore needed, which should include people with different impairment types, age and gender, and be used to improve policy and practice rapidly.

Plans and policies are being established in countries across the world to respond to COVID-19, including implementing partial or full lockdowns and increasing the capacity of the health system. These responses must be reviewed with respect to whether they are appropriate for and inclusive of disabled people. Ideally, this review should be done by or in partnership with disabled people. As an example, a key recommendation to protect from infection is to increase hand-washing. However, facilities may not be accessible for disabled people, or they may rely on carers for their hygiene maintenance. Messages should be tailored to include disabled people.

Crucially, plans must not violate the rights of disabled people, such as their right to equal access to healthcare, including ventilators. Plans must also not increase their vulnerability; as in the UK where the Coronavirus Bill explicitly suspends Care Act legislation, thereby reducing the responsibility on Local Authorities to meet care needs of disabled people ^16^. Inclusive planning is needed from the start, must be holistic and consistent, and have an available budget line to ensure that it can be made a reality. This is usually the responsibility of the Ministry of Health.

## The pandemic aftermath

At some point the world will emerge from the pandemic, and it is likely that millions of people will have been infected with COVID-19. Long-term consequences of infection are likely ^17^. People may be left with lasting lung damage or other physical conditions after recovery ^17^. Mental health conditions will almost certainly become more prevalent ^17^, particularly among healthcare personnel at risk of post-traumatic stress ^18^. People may have become newly disabled due to lack of healthcare (e.g. consequences of untreated ear infections). The impact of COVID-19 during pregnancy is not yet known, but there may be a higher prevalence of congenital anomalies among babies born to mothers who contracted the virus while pregnant. All these newly disabled people must have appropriate services provided. Livelihood and social assistance programmes are likely to be scaled up during and after the pandemic to cope with the economic consequences. These should particularly focus on disabled people as they work towards economic recovery, given their high vulnerability to poverty ^1^.

## Conclusion: Are there lessons from the UK about making the COVID-19 response disability-inclusive?

It is clear that disabled people are among the most vulnerable to contracting the virus and experiencing severe morbidity and mortality. The context of COVID-19 pandemic and its control will vary widely across the world. Although some good practice examples have emerged from the UK, these will need to be developed for relevance in different settings. There are, however, some commonalities. Additional efforts are needed to ensure that the response is disability-inclusive from the planning stage. This will require meaningful consultation with disabled people and their supporters, leadership at policy and programme level, and dedicated budget lines. Data collection on disability is needed to allow data disaggregation. The right to healthcare for disabled people is not negotiable, and must be protected within the COVID-19 pandemic.

## Data availability

### Underlying data

No data are associated with this article
