# Supplementary material for: Disability-inclusive COVID-19 response: What it is, why it is important and what we can learn from the United Kingdom’s response
Source: Wellcome Open Res. 2020 Apr 28;5:79. [Version 1] doi: 10.12688/wellcomeopenres.15833.1 (PMC7236579; doi:10.12688/wellcomeopenres.15833.1)
Supplement: Peer review history

# Peer review: Reviewer response for version 1

**DOI:** 10.21956/wellcomeopenres.17367.r38545

**Published:** 2020-05-18

**Authors:** Laura Nellums

**Affiliations:** 1 Division of Epidemiology and Public Health, School of Medicine, University of Nottingham, Nottingham, UK

Thank you for the opportunity to review this piece. I think this is a well written article, which highlights an important and underrepresented issue. I would support indexing of this article, and don't feel any significant amendments are needed.

 The article is clearly written, and strengthened by the inclusion of the distinct sections. The authors provide useful citations, including examples of guidance or best practice for disability-inclusive responses to the pandemic. The article provides useful information to guide our thinking and ultimately our response in the UK.

 The article could have been strengthened by additional recommendations regarding how to better include these populations, both within the UK and globally, particularly in light of limited financial resources, overstretched healthcare systems, overburdened staff, and a range of other marginalised or more vulnerable populations who must also have tailored responses to avoid inequities in access to care and health outcomes. It might also have been useful to provide more examples of the response to this population in other contexts, including in both high as well as low and middle income countries, and other examples of best practice or where significant disparities have emerged to better inform our response going forward.

Does the article adequately reference differing views and opinions?

Yes

Are all factual statements correct, and are statements and arguments made adequately supported by citations?

Yes

Is the Open Letter written in accessible language?

Yes

Where applicable, are recommendations and next steps explained clearly for others to follow?

Yes

Is the rationale for the Open Letter provided in sufficient detail?

Yes

Reviewer Expertise:

I am an Assistant Professor in Global Health, with particular expertise in disparities in access to care and health outcomes in marginalised populations.

I confirm that I have read this submission and believe that I have an appropriate level of expertise to confirm that it is of an acceptable scientific standard.

# Peer review: Reviewer response for version 1

**DOI:** 10.21956/wellcomeopenres.17367.r38543

**Published:** 2020-04-30

**Authors:** Helen Dickinson

**Affiliations:** 1 Public Service Research Group, School of Business, University of New South Wales, Canberra, Australia

This open letter argues that we need an effective disability-inclusive COVID-19 response in the face of the current pandemic. The evidence suggests that people with disabilities are often left behind in emergencies and there is a risk of this within the current pandemic. The letter seeks to outline what a disability-inclusive response would entail and what we can learn specifically from the UK government's response. 

 This is an important and well-written letter that draws attention to a number of issues that people with disability face in a pandemic context. It documents the points it makes well, providing evidence for these and sign posting a way forward somewhat. The only thing that was missing for me was mention of the well documented discrimination that many disabled people face - from broader society but also health services and practitioners. These exacerbate an already precarious position for many.

Does the article adequately reference differing views and opinions?

Yes

Are all factual statements correct, and are statements and arguments made adequately supported by citations?

Yes

Is the Open Letter written in accessible language?

Yes

Where applicable, are recommendations and next steps explained clearly for others to follow?

Yes

Is the rationale for the Open Letter provided in sufficient detail?

Yes

Reviewer Expertise:

Public administration; disability policy; health policy.

I confirm that I have read this submission and believe that I have an appropriate level of expertise to confirm that it is of an acceptable scientific standard.
